# Effects of Tatariside G Isolated from *Fagopyrum tataricum* Roots on Apoptosis in Human Cervical Cancer HeLa Cells

**DOI:** 10.3390/molecules190811145

**Published:** 2014-07-29

**Authors:** Yuan Li, Su-Juan Wang, Wei Xia, Khalid Rahman, Yan Zhang, Hao Peng, Hong Zhang, Lu-Ping Qin

**Affiliations:** 1 Department of Chinese Materia Medica, School of Pharmacy, Fujian University of Traditional Chinese Medicine, Fuzhou 350122, China; E-Mail: mengyingfeihen@126.com; 2 Central Laboratory, Shanghai Seventh People’s Hospital, Shanghai 200137, China; E-Mail: xhwang911@163.com; 3 Department of Nuclear Medicine, Shanghai Seventh People’s Hospital, Shanghai 200137, China; E-Mail: awingxia@163.com; 4 School of Pharmacy and Biomolecular Sciences, Faculty of Science, Liverpool John Moores University, Liverpool L3 3AF, England, UK; E-Mail: K.Rahman@ljmu.ac.uk; 5 Department of Pharmaceutical Botany, School of Pharmacy, Second Military Medical University, Shanghai 200433, China; E-Mail: jinhu7963@163.com; 6 Department of Traditional Chinese Medicine, Changhai Hospital, Second Military Medical University, Shanghai 200433, China

**Keywords:** natural product, cervical cancer, apoptosis, mitochondria, caspase

## Abstract

Cervical cancer is the second most common female carcinoma. Current therapies are often unsatisfactory, especially for advanced stage patients. The aim of this study was to explore the effects of tatariside G (TG) on apoptosis in human cervical cancer HeLa cells and the possible mechanism of action involved. An MTT assay was employed to evaluate cell viability. Hoechst 33258 staining and flow cytometry (FCM) assays were used to detect cell apoptosis. The protein expression of phosphorylated JNK, P38, ERK and Akt and cleaved caspase-3 and caspase-9 was evaluated by western blot analysis. Additionally, the mRNA expression of caspase-3 and caspase-9 was measured by fluorescent quantitative reverse transcription-PCR (FQ-RT-PCR). TG notably inhibited cell viability, enhanced the percentage of apoptotic cells, facilitated the phosphorylation of p38 MAPK and JNK proteins and caspase-3 and caspase-9 cracking, downregulated the phosphorylation level of Akt, and increased the loss of MMP and the mRNA expression of caspase-3 and caspase-9. TG-induced apoptosis is associated with activation of the mitochondrial death pathway. TG may be an effective candidate for chemotherapy against cervical cancer.

## 1. Introduction

Cervical cancer, characterized by the rapid and uncontrolled proliferation of cervical cells, is the second most common female cancer, with approximately 510,000 new cases and 280,000 deaths occurring around the world each year [1]. Although the mortality rate has declined over the years due to several advances in screening, diagnostic, prognostic and treatment modalities, the present treatments including chemotherapy, surgery and drugs are often unsatisfactory, especially in patients with advance stage cancer [2,3]. Thus, more effort has been directly focused on developing new drugs or therapeutics with few side effects to control cervical cancer.

One of the strategies is to consider natural products; these are plant-derived drugs and are considered to play an important role in treating cervical cancer due to their anticancer potential [4]. *Fagopyrum tataricum* (L.) Gaertn (tartary buckwheat), known as a medical and edible dual-purpose plant, mainly distributed in the north temperate zone, has been widely used as an important folk medicine in China for its antioxidant, antitumor, hypotensive, hypoglycemic, and hypolipidaemic effects [5,6]. Its seeds are rich in flavonoids, phenolics, high quality proteins and a well balanced amount of essential amino acids and minerals, which displays significant bioactivities of antioxidant and anti-inflammation properties. In addition, its roots are also an important medicinal part used for treating some stubborn diseases such as cancer and rheumatic disorders [7,8].

**Figure 1:**
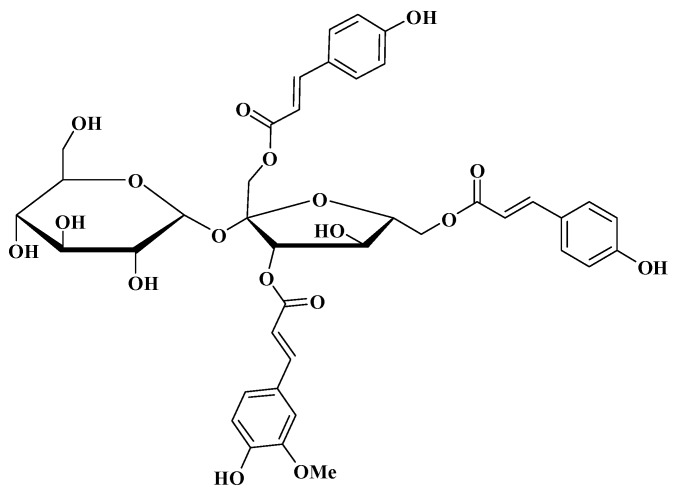
The chemical structure of tatariside G (TG).

Phenylpropanoid glycosides, which belong to flavonoid family, have been reported to display antitumor activity [9,10,11]. Tatariside G (TG, Figure 1) is a new phenylpropanoid glycosides compound isolated from the roots of *Fagopyrum tataricum* (L.) Gaertn, however, its antitumor activity has not been evaluated. In the present study, we observed the cytotoxic effects of TG in HeLa cells and explored the possible mechanism of action involved.

## 2. Results and Discussion

### 2.1. Effects of TG on HeLa Cells Viability

**Figure 2:**
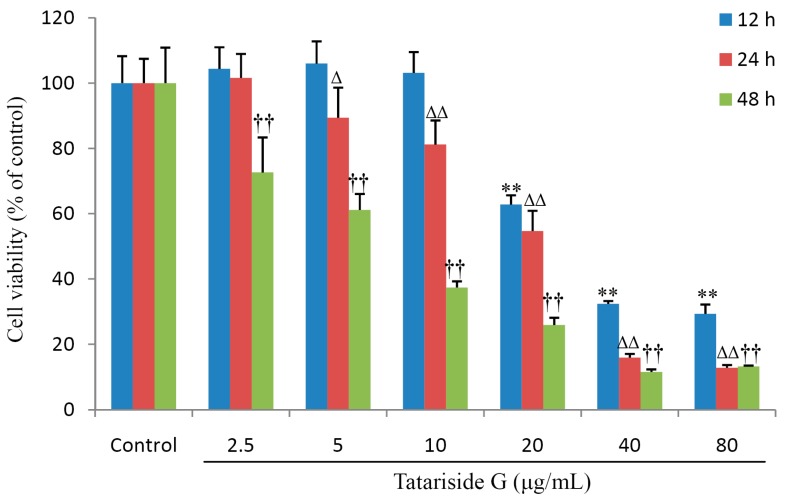
Effects of tatariside G on HeLa cells viability. After cells were treated with designated concentrations of tatariside G for 12, 24, and 48 h, respectively, cell viability was determined by MTT. Δ *p* < 0.05; **, ΔΔ, †† *p* < 0.01 compared with the control group. Data are expressed as the mean ± SD. N = 8.

As shown in Figure 2, cell viability was unchanged by the lower concentrations of TG (<10 μg/mL), but was significantly suppressed by the higher concentrations of TG (>20 μg/mL) 12 h post exposure to TG. Following exposure for 24 h, TG at a concentration of only 2.5 μg/mL did not exhibit the inhibitory activity in cells. Nevertheless cell viability evidently decreased in all treatment groups, when cells were treated with various concentrations of TG for 48 h. Reduction was more than 60% when TG was used at a concentration of 10 μg/mL. These results suggest that TG suppresses cell viability in a time- and dose-dependent manner.

### 2.2. TG Induces Apoptosis of HeLa Cells

**Figure 3:**
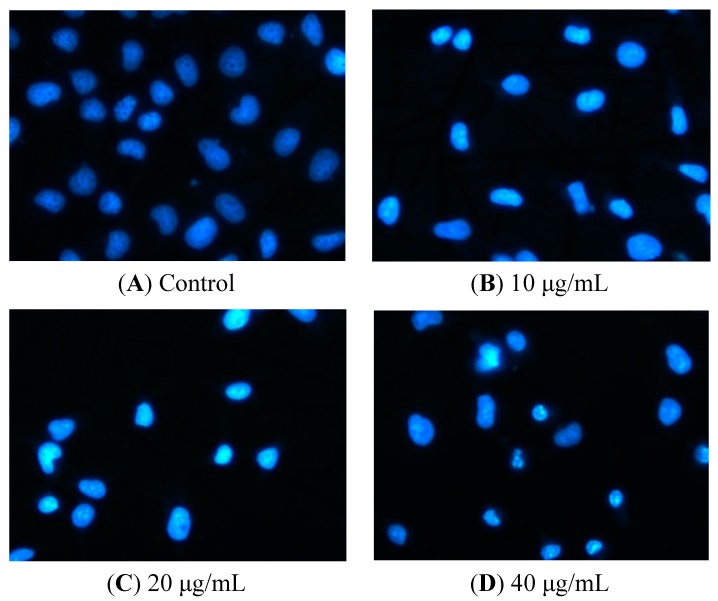
Nuclear morphologic changes in apoptotic HeLa cells by tatariside G. The cells were dyed with Hoechst 33258 after 12 h of treatment with tatariside G (10, 20, and 40 μg/mL). (**A**) Normal nuclei are symmetrical baby blue. (**B**) Intact nuclei are asymmetrical bright blue in the early stage of apoptosis. (**C**) and (**D**) Fragmented nuclei are presented in the advanced stage of apoptosis. Magnification: 400×.

DNA is cleaved into oligonucleosomal fragments once apoptosis occurs [12]. Hence following treatment with TG for 12 h, the nuclear morphological changes of cells were observed using Hoechst 33258 staining. Normal nuclei are symmetrical blue (Figure 3A), early in the apoptotic process the cells have an intact nucleus which is asymmetrical bright blue (Figure 3B) owing to chromatin aggregation, and in the next stage apoptotic cells have a fragmented nucleus (Figure 3C,D).

**Figure 4:**
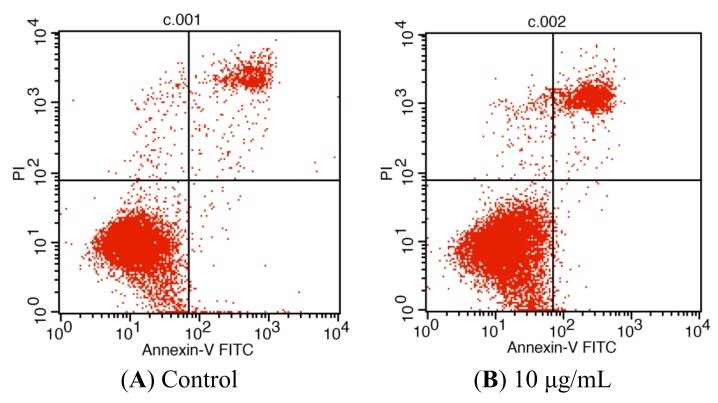

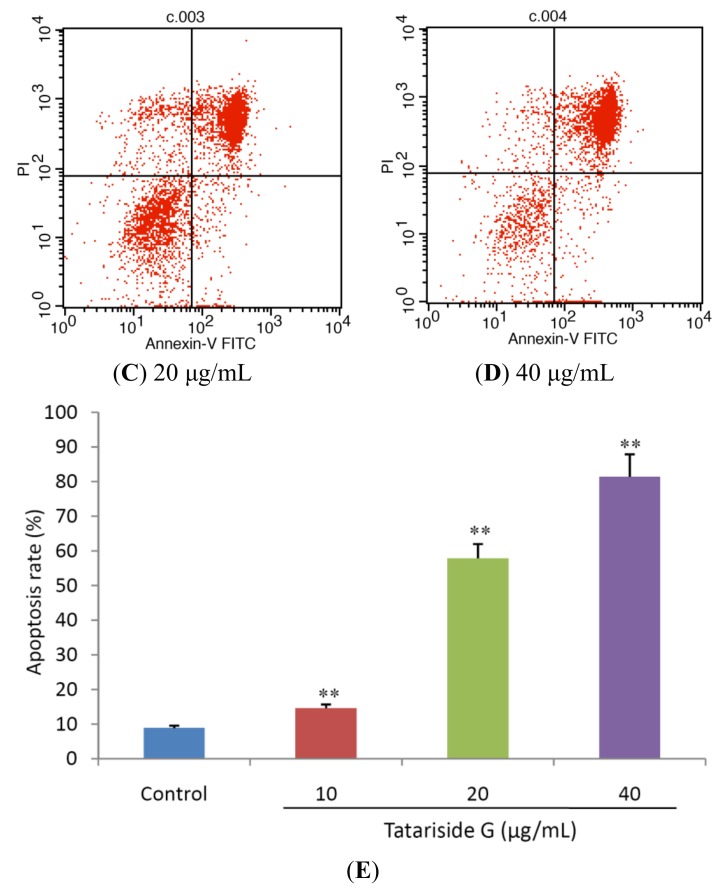
Tatariside G-induced apoptosis of HeLa cells was discriminated by AV and PI double staining. Following 24 h of treatment with tatariside G, the cells were labeled with AV and PI and analyzed by flow cytometry. The lower left quadrant shows vital cells (double negative) and the lower right quadrant indicates early apoptotic cells (AV positive but PI negative). The upper right quadrant represents late apoptotic cells (double positive). (**A**)–(**D**) Typical images. (**E**) Average apoptosis rate of cells in each group. ** *p* < 0.01 *vs.* the control group. Data are expressed as the mean ± SD. n = 3.

For relative quantification of cell apoptosis, we conducted a flow cytometry assay. As presented in Figure 4, most HeLa cells were vital in the control group (Figure 4A). However, after incubation with different concentrations of TG for 24 h (Figure 4B–D), the cells displayed a significant increase in the percentage of apoptosis (both AV and PI positive) in a dose-dependent manner from 8.85 ± 0.71% in the control up to 14.60 ± 1.08%, 57.87 ± 4.11%, and 81.40 ± 6.49%, respectively, in the treatment groups.

### 2.3. TG Decreases Mitochondrial Membrane Potential (MMP)

JC-1 is a lipophilic cation fluorescent dye that is capable of entering selectively into mitochondria when compared to rhodamines or other carbocyanines. Its colour changes reversibly from green to red with the increase of mitochondrial membrane potentials. Using a flow cytometer, green emission can be detected in fluorescence channel 1 (FL1) and red emission in channel 2 (FL2) and the ratio of red to green fluorescene intensity shows relative MMP of cells.

**Figure 5:**
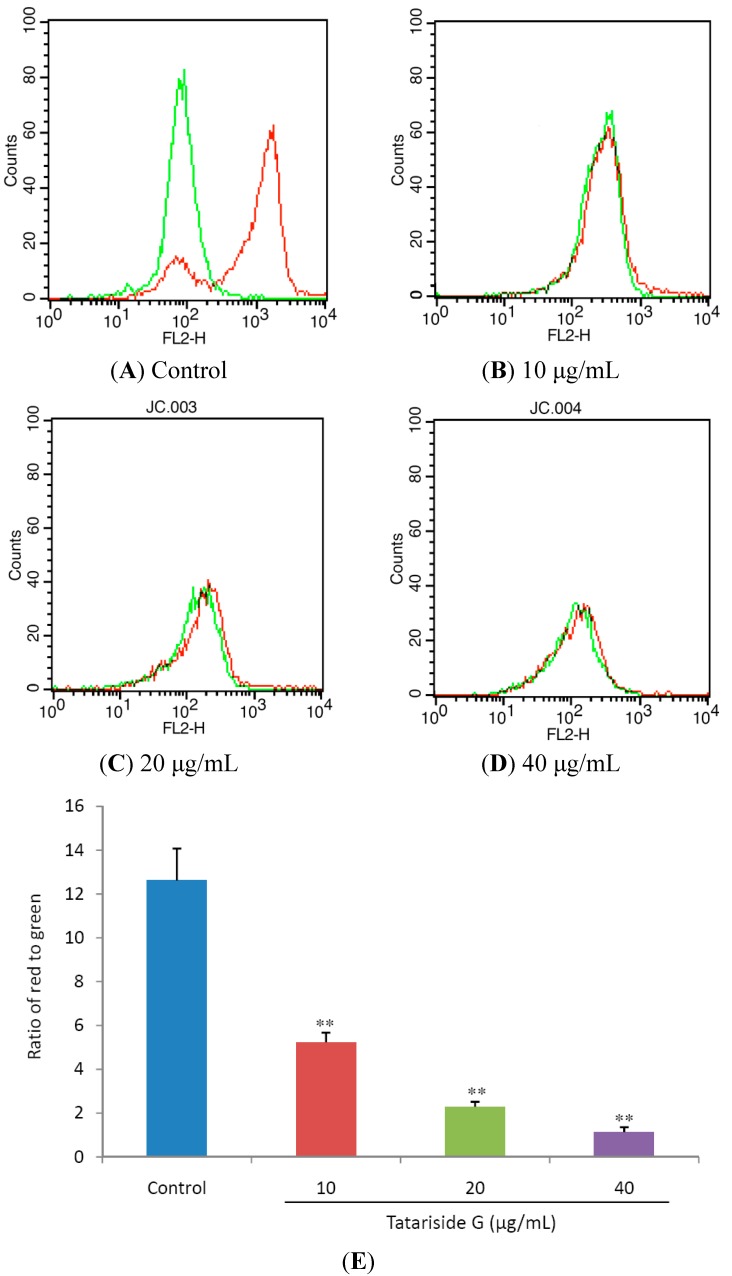
Effects of tatariside G on mitochondrial membrane potential (MMP). (**A**)–(**D**) Following treatment with tatariside G for 12 h, the fluorescent intensity of JC-1 in HeLa cells was determined respectively in FL1 and FL2. (**E**) MMP was estimated by the ratio of red to green fluorescene intensity. ** *p* < 0.01 *vs.* the control group. Data are expressed as the mean ± SD. n = 3.

MMP collapse is a pivotal step in the cell apoptosis process [13]. Figure 5A,B clearly show that, the red fluorescence intensity evidently decreased in the TG treated groups when compared with the control group. Figure 5C shows that the ratio of red to green was reduced significantly and dose‑dependently by 12 h of TG treatment. Taken together these results suggest that TG elicits the loss of MMP.

### 2.4. TG Regulates Protein Phosphorylation of p38, JNK, and Akt

**Figure 6:**
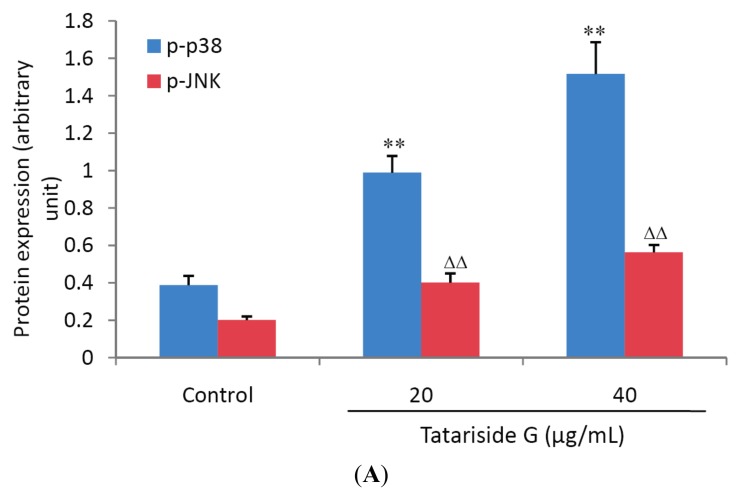

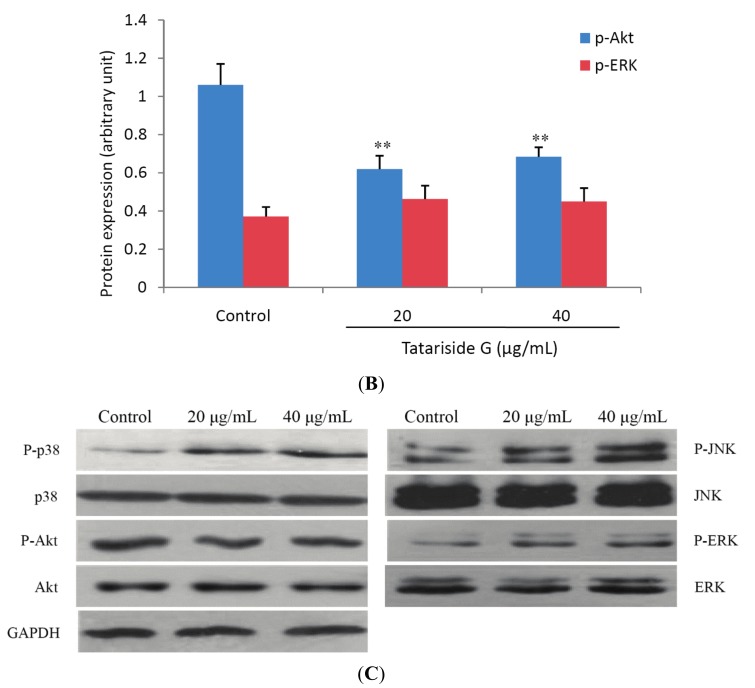
Effects of tatariside G on protein phosphorylation of p38, JNK, ERK and Akt. The relative expression levels of phosphorylated proteins were analyzed for p38 (**A**), JNK (**A**), ERK (**B**) and Akt (**B**). (**C**) Representative western blot picture of HeLa cells treated with tatariside G for 1 h. GAPDH was used as loading control. The results are presented as the ratio of phosphorylated to non-phosphorylated peptide intensities. **, ΔΔ *p* < 0.01 *vs.* the control group. Data are expressed as the mean ± SD. n = 3.

To elucidate the signal transduction pathways involved in TG-induced cell apoptosis, the expression of p38, JNK, ERK and Akt and the corresponding phosphorylated proteins, which are closely associated with cell apoptosis, was measured. Western blot analysis revealed that there was a significant increase in the phosphorylation levels of p38 and JNK (Figure 6A) but an evident decrease in the phosphorylation level of Akt (Figure 6B) after TG treatment for 1h. There was no significant change in the phosphorylation level of ERK protein (Figure 6B), indicating that TG facilitates cell apoptosis through both activation of p38 and JNK phosphorylation and inhibition of Akt phosphorylation.

### 2.5. TG Elevates the mRNA and Cleaved Protein Expression of Caspase-3 and -9

**Figure 7:**
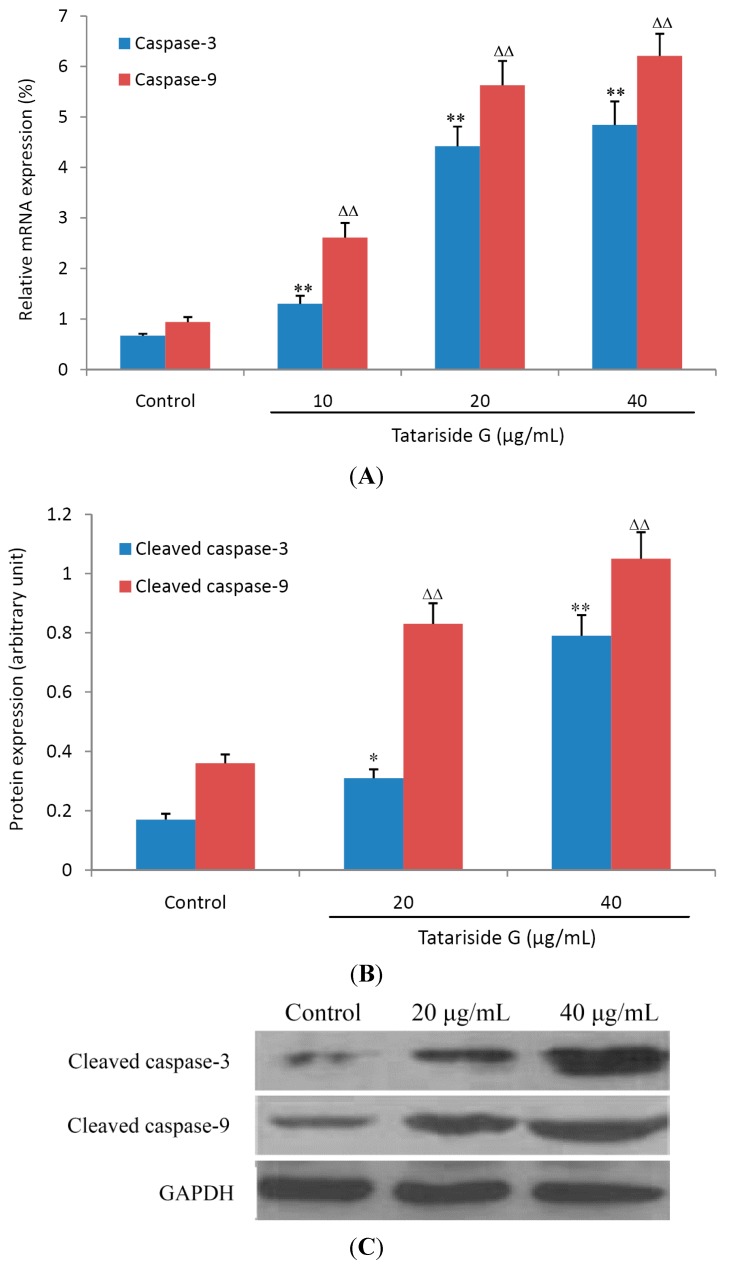
Effects of tatariside G on the mRNA and cleaved protein expression of caspase-9 and caspase-3. After treatment with tatariside G (10, 20, and 40 μg/mL) for 6 h or 12 h, the mRNA (**A**) and cleaved protein (**B**) expression of caspase-9 and caspase-3 was analyzed by FQ-RT-PCR or western blot. (**C**) Representative western blot picture of HeLa cells treated with tatariside G for 12 h. GAPDH was used as loading control. **, ΔΔ *p* < 0.01 *vs.* the control group. Data are expressed as the mean ± SD. n = 6 (**A**) or 3 (**B**).

Caspase-3 and -9 belong to a family of cysteine proteases and plays an important role in the process of executing cell apoptosis [14]. Caspase-3 can be activated by caspase-9 and cleaved into the active fragment. Figure 7 shows that TG treatment for 6 h (A) or 12 h (B) elevated the relative mRNA and cleaved protein expression of caspase-3 and caspase-9 in a dose-dependent manner, suggesting that TG induces cell apoptosis through triggering the caspase cascade response.

### 2.6. Discussion

Phytochemicals or plant extracts are gaining increasing attention due to their promising anticancer potential. It is well established that numerous cancer chemotherapeutic agents kill cancer cells by inducing apoptosis [3,15].

In the present study, we evaluated the growth inhibition and apoptosis-inducing effects of tatariside G (TG), a novel phenylpropanoid glycoside product isolated from the roots of *Fagopyrum tataricum* (L.) Gaertn, in human cervical cancer HeLa cells, and investigated the possible mechanisms of action involved.

TG evidently enhanced the inhibition of cell viability in a time- and dose- dependent manner, and caused chromatin aggregation and nuclear fragmentation. The flow cytometry assay showed that treatment with TG for 24 h elevated the percentage of apoptosis cells significantly and dose-dependently. These findings indicate that TG obviously induces apoptosis in HeLa cells.

Cell apoptosis (a cell programmed death) and the related signaling pathways have a profound impact on the development process of cancer. The apoptosis pathways mainly include two types, that is, the extrinsic death receptor pathway and the intrinsic mitochondrial pathway [16]. PI3K/Akt and MARK are major signal transduction pathways regulating cell apoptosis, proliferation, differentiation, and metastasis [17]. The MAPK system, including extracellular signal-regulated kinases (ERK), c-Jun N-terminal kinase (JNK) and p38 MAP kinase, relay extracellular signals to intracellular responses [18]. PI3K/Akt, by which survival signals are mainly transduced, is related to tumor cell resistance in both chemotherapy and radiation [18]. Both the MAPK and PI3K/Akt signal transduction systems can activate caspase-9 and caspase-3 by induction of mitochondrial membrane potential (MMP) collapse, subsequently leading to cells apoptosis occurring [19].

In order to clarify whether or not these signaling pathways are activated or inhibited, a western blot assay was performed to detect their phosphorylated protein expression. One h of TG treatment evidently enhanced the phosphorylation levels of both p38 MAPK and JNK, whilst the phosphorylation level of Akt was decreased. However, the phosphorylated protein expression of ERK was not significantly influenced by post 1 h treatment with TG. These results indicate that TG induces apoptosis of HeLa cells by simultaneous suppression of the Akt signal transduction pathway and activation of p38 and JNK signaling pathways.

For confirmation of TG-induced apoptosis by the intrinsic mitochondrial pathway, MMP was measured in HeLa cells. Following 12 h of treatment, MMP decreased abruptly and dose-dependently, suggesting activation of the mitochondrial apoptosis pathway. For further confirmation of this apoptosis, the mRNA and cleaved protein expression levels of caspase-9 and caspase-3 were measured by FQ-RT-PCR assay or western blot and it was found that after 6 h or 12 h of TG treatment, their relative mRNA and cleaved protein expression levels were elevated abruptly and dose-dependently.

Safety is vital to clinical uses of a drug, so we further evaluated the acute oral toxicity of TG, which did not cause any toxic effects as judged by the lack of fatalities in mice at a dose of 1.6 g/kg body weight (approximately 800 times of clinical dose), thus displaying good safety.

## 3. Experimental Section

### 3.1. Chemical and Reagents

Tatariside G (TG), which is a yellowish amorphous powder (molecular formula: C_40_H_42_O_18_; molecular weight: 828.27), was isolated from the ethyl acetate fraction of *Fagopyrum tataricum* roots as reported previously [20]. Stock solution was prepared by dissolving TG (5 mg) in DMSO (500 μL) and diluting to the desired concentrations with DMEM before utilization. MTT, DMSO, DMEM and 0.25% Trypsin were purchased from Sigma Chemical Co. (St. Louis, MO, USA), fetal bovine serum (FBS), phosphate buffered saline (PBS) and antibiotics (penicillin and streptomycin) from Beyotime Institute of Biotechnology (Haimeng, China), and Hoechst 33258 dye and JC-1 iodide from Invitrogen (Carlsbad, CA, USA). The AnnexinV-FITC apoptosis detection kit was obtained from JRDUN Biological Technology Co., Ltd. (Shanghai, China). Antibodies to Akt, p38, JNK, ERK and the corresponding phosphorylated proteins were the products of Cell Signaling Technology (Beverly, MA, USA). All reagents used were of analytical grade.

### 3.2. Cell Culture

HeLa cells were procured from JRDUN Biological Technology Co., Ltd., and were cultured in DMEM containing 10% FBS and 1% penicillin/streptomycin at 37 °C in a humidified atmosphere of 5% CO_2_ as described previously [21,22].

### 3.3. Determination of Cell Viability

Cell viability was evaluated by the MTT assay. HeLa cells were planted in a 96-well plate at a density of 1 × 10^5^ cells/mL and treated with designated concentrations of TG (2.5–80 μg/mL) for 12 h, 24 h, and 48 h, respectively. Subsequently, MTT (20 μL, 500 μg/mL) was added to each well and the plate was incubated for 4 h at 37 °C in 5% CO_2_ atmosphere. The supernatant was then aspirated carefully and DMSO (100 μL) was added to the well to lyse the formazan crystals. After 10 minutes, the absorbance was measured at 570 nm using an ELx-800 universal microplate reader (Bio-Tek, Winooski, VT, USA) and cell viability was expressed as a percentage of the value in the control group.

### 3.4. Nuclear Morphology Assay

The cells were incubated in a 6-well plate at a density of 2 × 10^5^/well for 24 h and were futher treated with TG at a concentration of 10, 20, and 40 μg/mL, respectively, for 12 h. Subsequently cells were harvested, washed twice with PBS, followed by fixation with 1 mL of 4% ice-cold paraformaldehyde for 10 min. 0.1% Triton was employed permeably for 20 min. After fixation with mixed glacial acetic acid and methanol (1:3), the cells were stained with 0.5 mL of 10 μM Hoechst 33258 (Sigma Chemical Co., St. Louis, MO, USA) at 37 °C in the dark for 5 min, then washed, observed and photographed under an inverted fluorescence microscope (DMI3000B, Leica, Wetzlar, Hessen, Germany) with the excitation and emission wavelengths of 340 and 460 nm, respectively. Apoptotic cells were defined based on nuclear morphology changes, such as chromatin condensation and fragmentation.

### 3.5. Annexin V-Propidium Iodide Apoptosis Detection

As previously described [13], the cell apoptosis rate was detected by flow cytometry using an Annexin V-FITC apoptosis detection kit (Nanjing Keygen Biotechnology Co. Ltd., Nanjing, China). After exposure to various concentrations of TG for 24 h, the cells were collected, washed with ice-cold PBS, and then re-suspended in 500 μL of binding buffer at a concentration of 1 × 10^6^ cells/mL. Subsequently, 5 μL of Annexin V-FITC was added for 10 min of incubation at room temperature. After addition of 5 μL of propidium iodide (PI), the cells were analyzed using a FACSCalibur flow cytometer (BD, San Jose, CA, USA).

### 3.6. MMP Measurement

To detect the mitochondria membrane potential (MMP), HeLa cells (5 × 10^5^ cell/mL) were seeded in a 6-well plate for incubation in the presence of TG (10, 20, 40 μg/mL) at 37 °C in 5% CO_2_ atmosphere for 12 h. Following the completion of treatment, cells were incubated with 5 μM JC-1 for 30 min at room temperature in the dark, washed twice with PBS to remove unbound dye, and then immediately analyzed by flow cytometry. Green fluorescence can be detected in fluorescence channel 1 (FL1) and red fluorescence in channel 2 (FL2). The ratio of red to green fluorescence intensity represents relative MMP.

### 3.7. Western Blot Analysis

Cells were incubated for 1 h or 12 h in the presence of designated concentrations of TG (20, 40 μg/mL), washed thrice with PBS, lysed in lysis buffer for 20 min, and centrifuged at 12,000 *g* for 10 min at 4 °C. The supernatant was stored at −80 °C until analyses. Protein concentrations were determined using BCA assay (Beyotime). An equal amount of proteins was subjected to electrophoresis on 12% SDS-polyacrylamide gel and transferred by electroblotting to polyvinylidene difluoride membrane (Millipore, Boston, MA, USA). Following blockage with 5% nonfat dry milk in PBS with 0.1% tween-20 for 1 h at room temperature, the membranes were probed with different primary antibodies overnight at 4 °C, subsequently washed, and incubated with 1:1000 dilutions of horseradish peroxidase-conjugated secondary antibodies for 1 h. The protein expression was detected using a chemiluminescence analyzer.

### 3.8. FQ-RT-PCR

The mRNA expression of caspase-3 and caspase-9 was detected by fluorescent quantitative reverse transcription-PCR (FQ-RT-PCR) in the HeLa cells treated with various concentrations of TG. In brief, following 6 h of treatment, the cells were washed with ice-cold PBS and mixed with degradation solution including Trizol and chloroform (0.2 mL of chloroform per 1 mL Trizol) for the cell lysates. After centrifugation, the collected aqueous phase was mixed with an equal volume of isopropanol to obtain total RNA and the isolated RNA was treated with RNasefree DNase (Promega, Beijing, China).

Reverse transcription was implemented with a cDNAsynthesis kit (Thermo, Waltham, MA, USA) according to the manufacturer’s instructions and as previously described [23]. PCR amplification was executed using a SYBR Green PCR kit (Thermo). Primer pairs for human genes were designed using the Primer Express Software (Applied Biosystems, Foster City, CA, USA) and are listed in Table 1.

Relative expression of mRNA (%) = 2^−ΔCT(1,2)^ × 100%, where CT represents threshold cycle, ΔCT1 = CT_(caspase-3)_ − CT_(GAPDH)_, ΔCT2 = CT_(caspase-9)_ − CT_(GAPDH)_. GAPDH served as a control.

**Table 1:** Primers used in FQ-RT-PCR analysis.

Gene	Primer sequence	Species	Amplicon size (bp)
Caspase-3	Forward: 5' AACTGGACTGTGGCATTGAG 3'	Human	161
	Reverse: 5' ACAAAGCGACTGGATGAACC 3'		
Caspase-9	Forward: 5' CCTCACCCTGCCTTATCTTG 3'	Human	189
	Reverse: 5' TCCCTCTTCCTCCACTGTTC 3'		
GAPDH	Forward: 5' CACCCACTCCTCCACCTTTG 3'	Human	110
	Reverse: 5' CCACCACCCTGTTGCTGTAG 3'		

### 3.9. Statistical Analysis

All results are presented as the mean ± SD. Statistical analysis was performed using a SPSS 13.0 statistical package and data are subjected to one-way analysis of variance (ANOVA), followed by Tukey’s test. Statistical significance was defined as *p* < 0.05.

## 4. Conclusions

TG, isolated from the roots of *Fagopyrum tataricum* (L.) Gaertn, elicits apoptosis of HeLa cells via the mitochondrial pathway, by triggering of p38 MAPK and JNK signaling, inhibition of Akt signal transduction, loss of MMP, and activation of caspase-9 and caspase-3. In this report TG-induced apoptosis has been confirmed for the first time. It may provide protection against cervical cancer, however the exact molecular mechanisms of action need further investigation.

## References

[1] Su J.H., Wu A., Scotney E., Ma B., Monie A., Hung C.F., Wu T.C. (2010). Immunotherapy for cervical cancer: Research status and clinical potential. Biodrugs.

[2] Ferguson P.J., Brisson A.R., Koropatnick J., Vincent M.D. (2009). Enhancement of cytotoxicity of natural product drugs against multidrug resistant variant cell lines of human head and neck squamous cell carcinoma and breast carcinoma by tesmilifene. Cancer Lett..

[3] Kim H.G., Song H., Yoon D.H., Song B.W., Park S.M., Sung G.H., Cho J.Y., Park H.I., Choi S., Song W.O., Hwang K.C., Kim T.W. (2010). *Cordyceps pruinosa* extracts induce apoptosis of HeLa cells by a caspase dependent pathway. J. Ethnopharmacol..

[4] Peng B., Hu Q., Liu X., Wang L., Chang Q., Li J., Tang J., Wang N., Wang Y. (2009). *Duchesnea* phenolic fraction inhibits *in vitro* and *in vivo* growth of cervical cancer through induction of apoptosis and cell cycle arrest. Exp. Biol. Med. (Maywood).

[5] Lin B., Hu C.L., Huang F., Han T. (2011). Research progress on chemical constituents and pharmacological effect of *Fagopyrum tataricum*. Drugs Clinic.

[6] Karki R., Park C.H., Kim D.W. (2013). Extract of buckwheat sprouts scavenges oxidation and inhibits pro-inflammatory mediators in lipopolysaccharide-stimulated macrophages (RAW264.7). J. Integr. Med..

[7] Guo Z.J. (2003). Shanxi Qi Yao.

[8] Guo Z.J., Bu X.X., Wang J.X., Lv J.X. (2006). Plant resources and research summary on “Shanxi Qi Yao”. Chin. J. Ethnomed. Ethnopharm..

[9] Brown L.L., Larson S.R., Sneden A.T. (1998). Vanicosides C–F; new phenylpropanoid glycosides from *Polygonum pensylvanicum*. J. Nat. Prod..

[10] Kuo Y.H., Hsu Y.W., Liaw C.C., Lee J.K., Huang H.C., Kuo L.M. (2005). Cytotoxic phenylpropanoid glycosides from the stems of *Smilax china*. J. Nat. Prod..

[11] Takasaki M., Konoshima T., Kuroki S., Tokuda H., Nishino H. (2001). Cancer chemopreventive activity of phenylpropanoid esters of sucrose, vanicoside B and lapathoside A from *Polygonum lapathifolium*. Cancer Lett..

[12] Saraste A., Pulkki M.K. (2000). Morphologic and biochemical hallmarks of apoptosis. Cardiovasc. Res..

[13] Wu J.G., Ma L., Zhang S.Y., Zhu. Z.Z., Zhang H., Qin L.P., Wei Y.J. (2011). Essential oil from rhizomes of *Ligusticum chuanxiong* induces apoptosis in hypertrophic scar fibroblasts. Pharm. Biol..

[14] Thornberry N.A. (1998). Caspases: Key mediators of apoptosis. Chem. Biol..

[15] Mandal S.K., Biswas R., Bhattacharyya S.S., Paul S., Dutta S., Pathak S., Khuda-Bukhsh A.R. (2010). Lycopodine from *Lycopodium clavatum* extract inhibits proliferation of HeLa cells through induction of apoptosis via caspase–3 activation. Eur. J. Pharmacol..

[16] Wajant H. (2002). The Fas signaling pathway: More than a paradigm. Science.

[17] Cargnello M., Roux P.P. (2011). Activation and function of the MAPKs and their substrates; the MAPK–activated protein kinases. Microbiol. Mol. Biol. Rev..

[18] Fresno V.J., Casado E., de Castro J., Cejas P., Belda-Iniesta C., Gonzalez-Baron M. (2004). PI3K/Akt signalling pathway and cancer. Cancer Treat. Rev..

[19] Sun X.M., MacFarlane M., Zhuang J., Wolf B.B., Green D.R., Cohen G.M. (1999). Distinct caspase cascades are initiated in receptor-mediated and chemical-induced apoptosis. J. Biol. Chem..

[20] Zheng C.J., Hu C.L., Ma X.Q., Peng C., Zhang H., Qin L.P. (2011). Cytotoxic phenylpropanoid glycosides from *Fagopyrum tataricum* (L.) Gaertn. Food Chem..

[21] Zheng G.Y., Qin L.P., Yue X.Q., Gu W., Zhang H., Xing H.L. (2014). Portulacerebroside A induces apoptosis via activation of the mitochondrial death pathway in human liver cancer HCCLM3 cells. Phytochem. Lett..

[22] Ghosh S., Bishayee K., Paul A., Mukherjee A., Sikdar S., Chakraborty D., Boujedaini N., Khuda-Bukhsh A.R. (2013). Homeopathic mother tincture of *Phytolacca decandra* induces apoptosis in skin melanoma cells by activating caspase-mediated signaling via reactive oxygen species elevation. J. Integr. Med..

[23] Zhang H., Yu C.H., Jiang Y.P., Peng C., He K., Tang J.Y., Xin H.L. (2012). Protective effects of polydatin from *Polygonum cuspidatum* against carbon tetrachloride–induced liver injury in mice. PLoS One.

